# Editorial: Additive Manufacturing Applications in Cardiovascular Research

**DOI:** 10.3389/fbioe.2022.970932

**Published:** 2022-07-12

**Authors:** Fatemeh Kabirian, Nooshin Haghighipour, Petra Mela, Ruth Heying

**Affiliations:** ^1^ Cardiovascular Developmental Biology, Department of Cardiovascular Sciences, KU Leuven, Leuven, Belgium; ^2^ National Cell Bank of Iran, Pasteur Institute of Iran, Tehran, Iran; ^3^ Medical Materials and Implants, Department of Mechanical Engineering and Munich Institute of Biomedical Engineering, TUM School of Engineering and Design, Technical University of Munich, Garching, Germany

**Keywords:** additive manufacturing, 3D printing, bioprinting, cardiovascular models, 4D printing, cardiovascular implants

Additive manufacturing (AM) applications enabled remarkable progress in the cardiovascular field during the last decades. 3D printing has become widely accessible and the articles published in this research topic nicely represent the range of different approaches benefiting from this technology, from cardiovascular biomedical engineering to education in cardiovascular medicine.

AM is a layer-by-layer manufacturing process with the ability to create complex geometries according to computer-aided design (CAD) data. It is widely used for the fabrication of customized medical devices and drug delivery systems in the field of biomedical engineering. While cardiovascular research has shown interesting progress in the development of cardiovascular scaffolds, the application of AM can address remaining limitations In detail, the capability to fabricate complex structures with controlled architecture and to position cells with unprecedented spatial accuracy enables the fabrication of tissue/organ substitutes ideally recapitulating the mechanical and biological properties of the original, healthy structures.

The growing prevalence of cardiovascular diseases is leading to an increased need of cardiovascular surgeries such as coronary artery bypass graft surgery, angioplasty and valve replacement. Continuous progress in the field includes advancements in material’s design and fabrication strategies. An interesting approach was presented by Song et al. who used yak pericardium as a promising material for the development of transcatheter heart valves. The yak pericardium possessed an appropriate thickness and mechanical properties and showed a lower calcification and inflammation response when implanted subcutaneously in a rat model compared to two different bovine pericardial tissues.

Despite the advantages of natural materials there is a big demand for synthetic grafts. 3D printing offers the possibility to fabricate reproducible and accurate synthetic grafts which can even become programmable and adaptable after implantation by using 4D printing with smart biomaterials.

3D models have become popular as a tool contributing to clinical diagnosis and treatment. Printed on the basis of patients’ CT data, 3D printed models resemble the individual cardiovascular anatomy and enable a careful planning of surgical and minimally invasive interventions, and, therewith, facilitate the decision-making process of surgeons and cardiologists towards the best therapy for the individual patient. In congenital heart disease, the most common indications for clinical use of 3D printed models include complex forms of double outlet right ventricle and transposition of the great arteries, anomalous systemic and pulmonary venous connections, and heterotaxy. Gómez Ciriza et al. represents this approach by summarizing their experience and the advantages of 3D printed models and commented on the rapid preparation process and the cost-effectiveness. Over 7-years, 138 models from different congenital heart disease patients were fabricated. The average production cost per model was 85.7 Euro and designing and fabrication times were 136 min and 13.5 h, respectively.

Another relevant application of 3D printed models is the use in teaching and training of medical students in the cardiovascular field. These models allow a visual learning, which is not achievable *via* other platforms. Yoo et al. summarized their constructive experience in preoperative surgical simulation. Hands-on surgical training (HOST) is an educational approach for surgical trainee, especially for congenital heart operations, using 3D printed models. The authors concluded that 3D models are valuable for development of the surgical entry level skills such as cutting, suturing and cannulating. In addition, the step-by-step practice of surgical procedures from basic to complex elements is possible and 3D printed models can resemble the tissue features as e.g. the myocardial thickness. Also, 3D models for cardiac valve operations are under development.

Future directions evolving towards 4D printing have been summarized by Kabirian et al., highlighting the potential of combining 3D printing technology and smart materials to fabricate implants changing their shape, function, and properties over time. In the cardiovascular field, 4D printing can be used to produce programmable prostheses that react to specific stimuli to facilitate implantation and *in vivo* functionality. For instance, 4D printed cardiac patches are designed to be adjustable to the specific cardiac architecture and are implantable using a minimal invasive approach via two small incisions. 4D printed customized and biodegradable left atrial appendage occluders (LAAOs) are a new class of LAAOs with improved *in vivo* performance minimizing the device associated risks of commercial nitinol occluders. Self-expandable vascular stents and grafts have also been created by 4D printing and are advantageous compared to the commercially available vascular grafts which are limited by fixed dimensions and mechanical properties. Thereby, 4D printing opens new horizons in vascular surgery.

Drug delivery systems can also be incorporated into 3D printed cardiovascular implants to improve, for example, hemocompatibility and bacterial resistance. At the same time, 3D *in vitro* models with tissue-specific cell-cell and cell-matrix interaction are a powerful tool to study cardiovascular disease onset and progression. They can also be used for direct drug testing on patient-specific cells, especially in combination with microfluidic systems to recapitulate (patho) physiological hemodynamic load conditions.

In conclusion, the presented applications of AM demonstrate the important role of this technology in promising future development of the cardiovascular field.

